# Machine learning predictive model for evaluating the cooking characteristics of moisture conditioned and infrared heated cowpea

**DOI:** 10.1038/s41598-022-13202-4

**Published:** 2022-06-02

**Authors:** Opeolu. M. Ogundele, Ayooluwa. T. Akintola, Beatrice M. Fasogbon, Oluwafemi.A. Adebo

**Affiliations:** Department of Biotechnology and Food Technology, Faculty of Science, University of Johannesburg, Doornfontein Campus, P.O. Box 17011, Gauteng, South Africa

**Keywords:** Biotechnology, Plant sciences, Mathematics and computing

## Abstract

Cowpea is widely grown and consumed in sub-Saharan Africa because of its low cost and high mineral, protein, and other nutritional content. Nonetheless, cooking it takes considerable time, and there have been attempts on techniques for speeding up the cooking process without compromising its nutritious value. Infrared heating has recently been proposed as a viable way of preparing instantized cowpea grains that take a short amount of time to cook while maintaining desired sensory characteristics. Despite this, only a few studies have shown the impact of moisture, temperature, and cooking time on cooking characteristics such as bulk density, water absorption (WABS), and the pectin solubility of infrared heated cowpea precooked using this technology. Artificial neural network was used as a machine learning tool to study the effect of a prediction model on the infrared heating performance and cooking characteristics of precooked cowpea seeds. With R values of 0.987, 0.991, and 0.938 for the bulk density, WABS, and pectin solubility, respectively, the prediction model created in this study utilizing an artificial neural network (a type of machine learning) outperformed the traditional linear, 2-factor interaction, and quadratic models.

## Introduction

Cowpea (*Vigna unguiculata*) is among the leguminous crop mostly cultivated in Sub-Saharan Africa^1^. It is an affordable and sustainable plant source of protein, phytonutrients, and minerals, useful in combating protein-energy malnutrition and food insecurity^2^. Cowpea remains a vital raw material used in making traditional dishes in various forms such as couscous (*shô basi*), fritters (*akara*), steamed pudding (*moimoi*)^3,4^. Cowpea is mainly consumed by cooking the whole grains in water for about 2 h, however this long processing time and as a result high energy consumption during food preparation remains the major limitation to its consumption in urban areas^3^. Several methods including soaking, precooking, and usage of alkaline salts treatments have been useful to decrease cooking time and enhancing the nutritional quality of pulses^5–7^. Recently, infrared heating, a novel thermal food processing technique has also been reported to significantly improved cooking characteristics of pulses such as cowpea^8^.

Infrared radiation heating uses electromagnetic radiation in the infra-red region within the wavelength of about 3 to 1000 µm to generate heat^9^. Infrared technology has been useful in many foods manufacturing processes, including drying, boiling, heating, roasting of food, cooking food and sterilization grains^10^. It is an environmentally friendly thermal treatment and considered as an advanced thermal process that can be exploited in food processing. Different studies on infrared heating of cowpea grains have shown that the combination of varying moisture level, infrared radiation temperature and time could reduce its cooking time by more than 50%, improve sensory properties including appearance and color and protein quality of the infrared treated cowpea grains^3,8,11,12^. These processing conditions causes an increase in solubility of pectin responsible for rapid water uptakes, separation of cells along the cell wall and other physicochemical and structural changes of starch and protein as a result facilitated softening of cowpeas and reduction in the cooking time^8,11^. Developing an optimization model has been useful in predicting and optimizing the parameters in grain processing to improve the pulses quality for instance cowpea grains^8,13^.

Response surface methodology (RSM) is one of the utilized conventional optimization model techniques in pulses processing that has been useful in predicting and optimizing processing parameters such as germination^14^, drying^15^, extrusion^16^ and infrared heating^8,13^ of pulses to improve quality and developing new products. However, the performance of models developed using RSM is limited. Recently, with the advent of the 4^th^ industrial revolution, artificial neural network (ANN) has been considered as a more efficient tool for model predictions of biodiesel production^17^ and even in food processing^18^. ANN is a mathematical algorithm which has the capability of relating the input and output parameters, learning from examples through iteration, without requiring a prior knowledge of the relationships of the process parameters^18^. ANN models have been used for process control in thermal food processing including modelling tool in several food processing applications like drying^19,20^, heat transfer and thermal process predictions^18,21^. Furthermore, artificial intelligence is one of the approaches which has proven to be efficient with improving the processing quality of grains like wheat in the development of food products^22,23^. Application of ANN for food processes such as legume processing have been reported^24,25^. Nonetheless, to the best of our knowledge, there is still a dearth of information available on predicting the properties of pulses prepared by infrared heating using artificial intelligence, which is critical for improving the cooking characteristics of pulses such as cowpea, as an important source of protein. Therefore, this study aimed at developing a predictive model for evaluating the cooking characteristics of cowpea (specifically bulk density, water absorption capacity and pectin solubility) under varied processing parameters of moisture content, temperature, and time within a closed system of an infrared heater.

## Materials and methods

### Materials

Cowpea (*Vigna unguiculata:* Agrinawa variety) seeds were received from the South African Agricultural Research Council's Institute for Tropical and Subtropical Crops in Nelspruit, South Africa. The seeds were manually selected to remove faulty seeds and kept at 4 °C until moisture preconditioning, and infrared heat treatment were done.

### Experimental design and sample preparation

**Figure 1:**
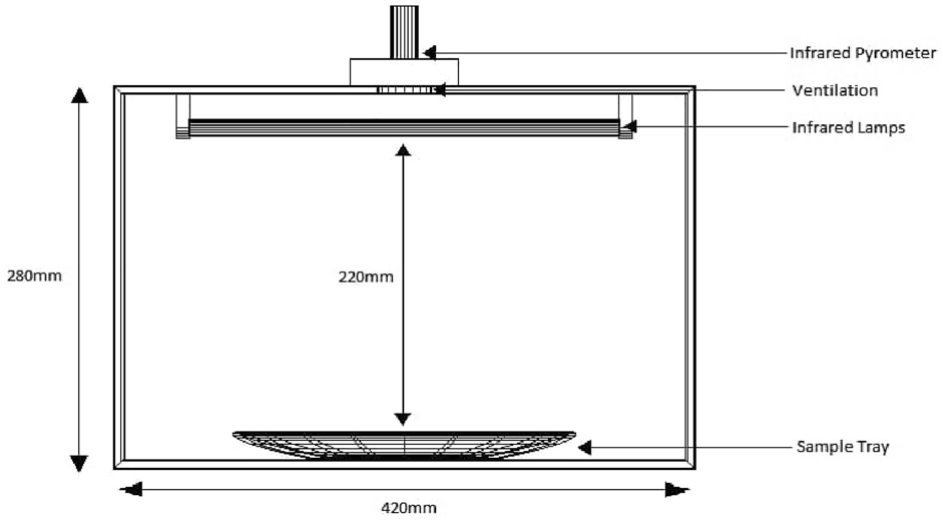
Schematic diagram of the infrared heating system.

Using Statistica version 7 statistical software, a series of experiments were statistically constructed based on RSM-central composite design (CCD) (StatSoft, Tulsa, USA). Level of moisture, infrared heating temperature, and time were the independent variables of pre-treatments investigated, with intervals of 32–57%, 114–185 °C, and 2–18 min, respectively (Table 1). The selection of the parameter levels was based on other studies in the literature on the production of infrared heated cowpea seeds^3,8,11,12^. Bulk density, WABS, and pectin solubility were the dependent variables of pretreatments assessed in this study. The combination of variables resulted in the creation of fifteen (15) experimental runs, each of which was carried out in triplicate, resulting in the generation of 45 rows of experimental data (Table 2). The cowpea seeds were first soaked in water to attain 32, 40, 45, 54, and 57% moisture (dry basis) using the method reported by Mwangwela, Waniska^11^ and infrared heated using a closed system infrared heater power output 3KW (MW184, Delphius Technologies, Pretoria, South Africa) for each experimental run (Table 2). A schematic diagram of the infrared heating system used in this study is shown in Fig. 1. It has three infrared emitters (Quartz tube infrared emitters) with power outputs ranging between 0 to 3 KW, short wave infrared with a wavelength peak emission at 2.9 µm. The samples produced were maintained at 4 °C in an airtight container until further analysis.

**Table 1:** Descriptive Property of Raw Data.

Properties	Predictors			Actual responses		
	Moisture (%)	Temp (°C)	Time (min)	Bulk-density (g/kg)	WABS (%)	Pectin Solubility (%)
Minimum	32.65	114.72	2.00	0.59	95.01	146.79
Maximum	57.34	185.27	18.58	0.67	128.95	253.23
Median	45.00	150.00	8.00	0.62	108.67	180.64
Mean	46.06	150.00	8.70	0.63	111.46	189.02
Standard Deviation	6.96	19.69	5.17	0.02	12.09	27.29
Standard Error	1.03	2.93	0.77	0.00	1.80	4.06

The summary in Table 1. is for the 45 numbers of experiments (15*3replicates).

**Table 2:** Experimental raw input data.

S/N	Moisture	Temperature	Time	Bulk density	WABS	Pectin Solubility
	(%)	(°C)	(min)	(g/kg)	(%)	(%)
1	40.00	130.00	2.00	0.66	127.63	180.75
2	40.00	130.00	2.00	0.66	125.18	177.42
3	40.00	130.00	2.00	0.66	123.81	179.73
4	40.00	130.00	14.00	0.64	115.60	167.11
5	40.00	130.00	14.00	0.64	114.38	164.07
6	40.00	130.00	14.00	0.64	113.98	165.08
7	40.00	170.00	2.00	0.65	128.71	161.34
8	40.00	170.00	2.00	0.65	125.31	164.38
9	40.00	170.00	2.00	0.66	126.45	163.14
10	40.00	170.00	14.00	0.62	97.50	190.76
11	40.00	170.00	14.00	0.62	95.96	190.15
12	40.00	170.00	14.00	0.61	96.00	190.47
13	54.00	130.00	2.00	0.62	128.95	170.74
14	54.00	130.00	2.00	0.61	126.22	171.65
15	54.00	130.00	2.00	0.62	125.14	170.52
16	54.00	130.00	14.00	0.63	98.40	221.09
17	54.00	130.00	14.00	0.63	100.39	224.12
18	54.00	130.00	14.00	0.63	101.09	223.22
19	54.00	170.00	2.00	0.61	126.06	178.02
20	54.00	170.00	2.00	0.61	126.00	182.27
21	54.00	170.00	2.00	0.61	125.49	180.89
22	54.00	170.00	14.00	0.59	103.97	208.35
23	54.00	170.00	14.00	0.59	101.09	213.20
24	54.00	170.00	14.00	0.60	102.11	211.73
25	45.00	150.00	8.00	0.62	104.16	227.76
26	45.00	150.00	8.00	0.61	102.00	229.58
27	45.00	150.00	8.00	0.62	103.96	228.59
28	32.65	150.00	8.00	0.67	116.66	174.08
29	32.65	150.00	8.00	0.66	118.16	197.13
30	32.65	150.00	8.00	0.66	119.36	187.14
31	57.34	150.00	8.00	0.61	95.01	172.56
32	57.34	150.00	8.00	0.60	95.31	173.78
33	57.34	150.00	8.00	0.61	96.00	173.91
34	45.00	114.72	8.00	0.64	127.08	146.79
35	45.00	114.72	8.00	0.63	124.67	148.91
36	45.00	114.72	8.00	0.64	125.10	147.65
37	45.00	185.27	8.00	0.60	102.78	248.99
38	45.00	185.27	8.00	0.59	104.65	253.23
39	45.00	185.27	8.00	0.60	104.07	249.88
40	45.00	150.00	18.58	0.61	98.01	179.54
41	45.00	150.00	18.58	0.61	99.60	193.49
42	45.00	150.00	18.58	0.61	98.02	180.22
43	45.00	150.00	8.00	0.62	111.46	179.24
44	45.00	150.00	8.00	0.62	105.67	182.57
45	45.00	150.00	8.00	0.62	108.67	180.64

#### Analyses

Bulk density of cowpea seed samples was determined using the method described by Alves, Da Silva et al.^26^. Water absorption capacity of cowpea seed samples was determined using the method described by Ogundele and Emmambux^27^. Soluble pectin, hot water-soluble pectin (HWSP) of cowpea seed samples was determined as described by Ndungu Emmambux and Minnar^12^. The experimental data obtained from the analyses are presented in Table 2.

### Artificial neural network (ANN) modeling

**Figure 2:**
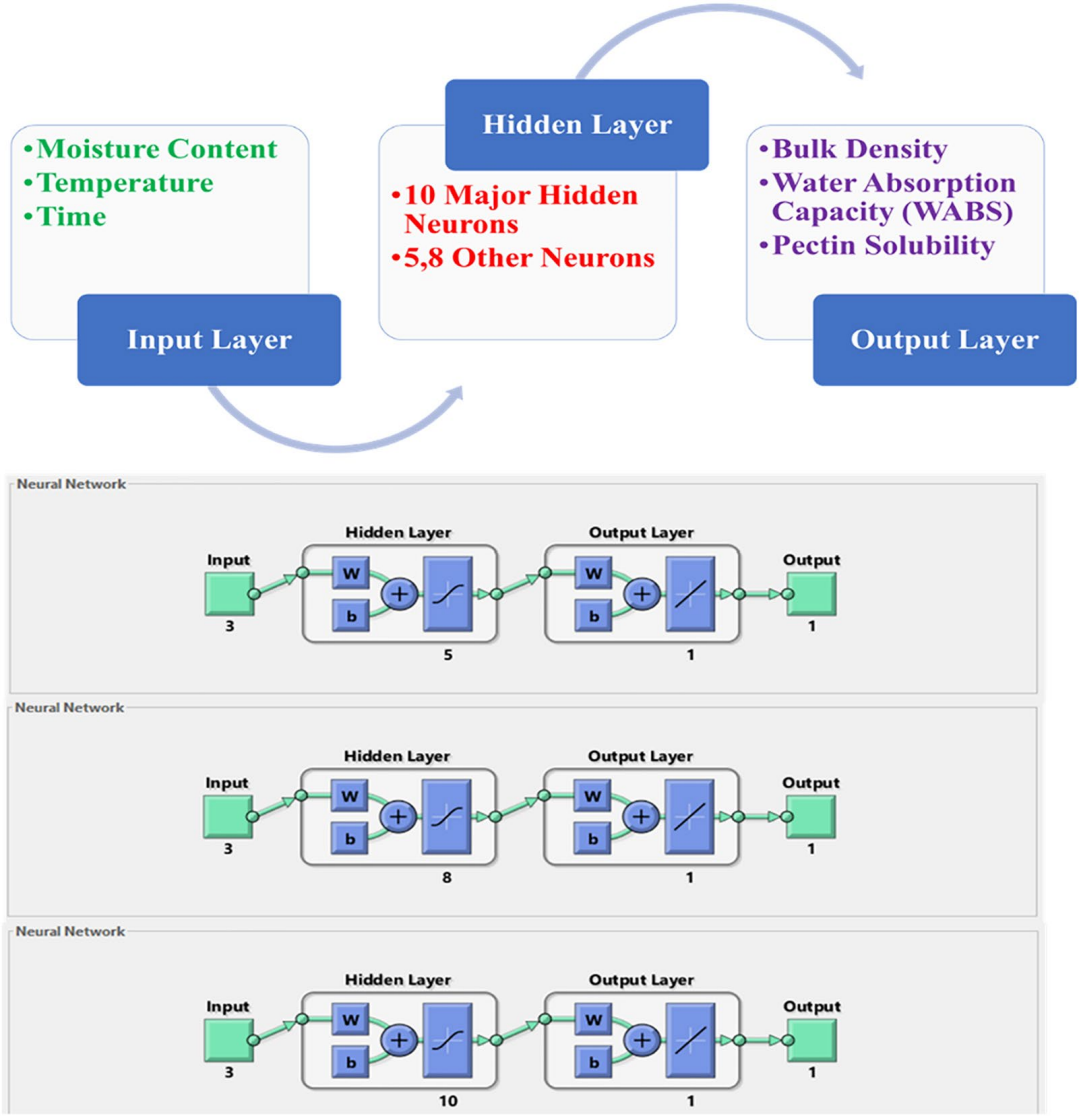
The structure of ANN utilized in the study.

ANN is a supervised form of machine learning driven by the availability of dataset, where raw dataset is the input into the neural system used in developing a predictive model, and subsequently a set of predicted outputs. The neural system consists of hidden layers of neurons that are useful in learning the patterns specific to the raw data and assists in producing possible related outputs^28,29^. A simple representation of the neural network structure used in our paper is presented in Fig. 2. Differences between the output value in the raw data and the new set of output produced after subjecting our data to ANN algorithm results in an error^29^.

### Data cleaning

The raw data was cleaned and utilized as the input for machine learning prediction using ANN. The predictive variables are moisture temperature and time, while the response variables are bulk density, WABS, and pectin solubility (Table 1). The descriptive property of the raw data is presented in Table 1, while the raw data used as input is presented in Table 2.

### Data processing

The processing of data using ANN includes data training, data validation, and data testing. For this research work, MATLAB software was used for ANN modeling. The algorithm used for data processing is the Levenberg–Marquardt algorithm, owing to its swiftness and steadiness in convergence^30^. For data training, the raw data is given as input to the neural network system. During this stage, it fine-tunes the data in retrospect to the error produced. The validation process estimates network’s generalization, besides, signifies when the training process should stop once there is no more progress in data generalization, lastly, data testing evaluates and offers a stand-alone measure of the network’s performance before, during and after data training. For data training, validation, and testing, the dataset was rationed in 70, 15, and 15% respectively. 10 hidden neurons were used for deriving the best predictive model for bulk density and WABS, while three neurons (5,8, and 10) were used for pectin solubility (Fig. 2). This was to produce improved predictive model for pectin solubility.

### Performance of predictive model

Understanding the performance of a model is key to improve prediction accuracy. The performance indicators used to evaluate the accuracy of the predictive model developed in this work includes mean square error (MSE), coefficient of correlation (R) and coefficient of determination (R^2^). Besides, the performance of the ANN model developed was compared with the performance of conventional models such as linear, two factor interaction (2FI), quadratic and cubic model using R^2^. This was to affirm the accuracy of utilizing ANN for developing a predictive model for estimating the bulk density, WABS, and pectin solubility of cowpea precooked using infrared heating.

### Ethical guideline statement

The author (s) declare the plant material used in this research complied with relevant institutional, national, and international guidelines and legislation.

## Result and discussion

**Figure 3:**
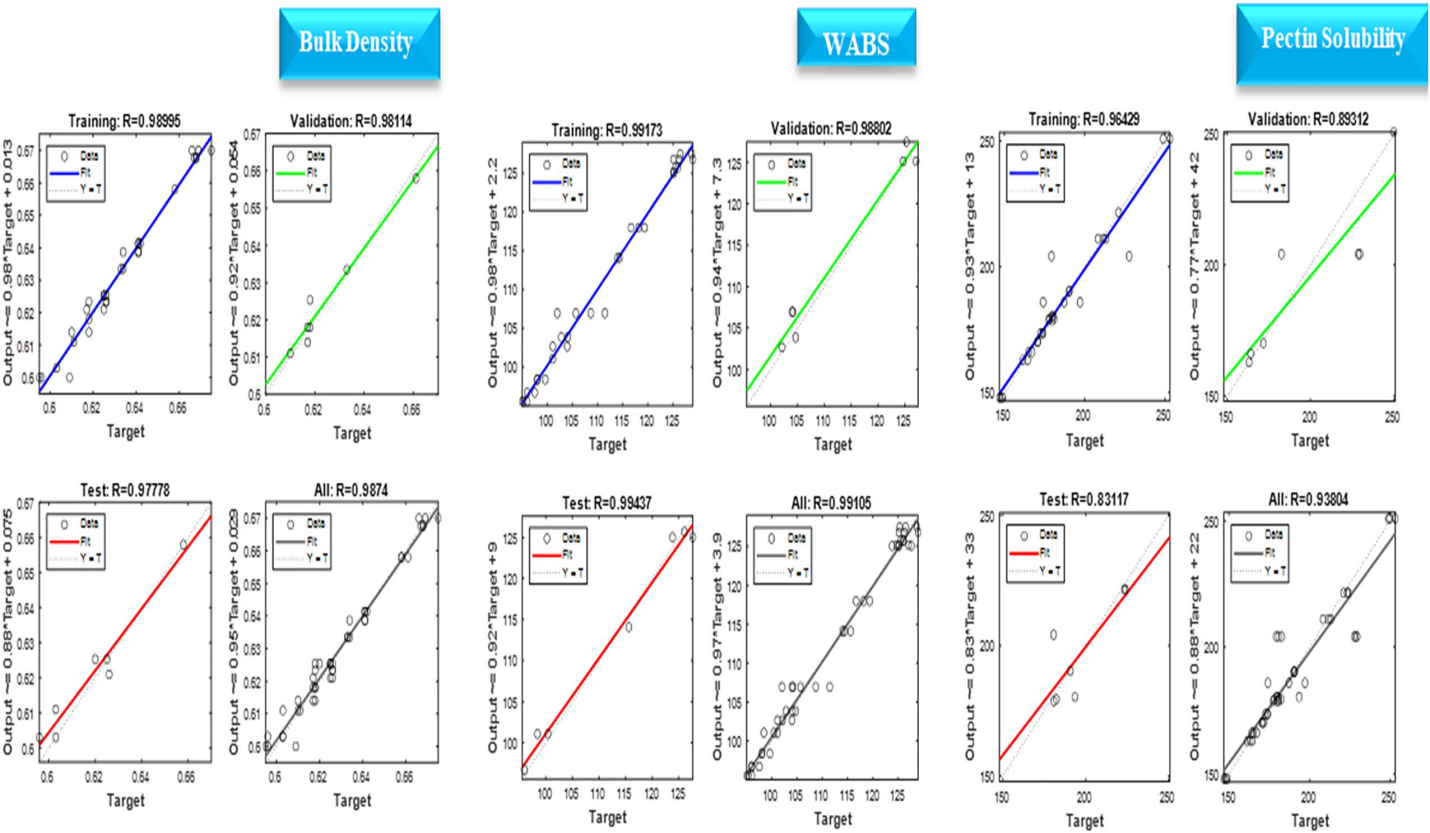
Significant Models with their R-Value.

A predictive model was developed using ANN for the bulk density, WABS and pectin solubility of cowpea precooked using infrared heating. Three main factors that influence the response were fed into the neural network as the independent variables, the experimental data of the process was cleaned and processed using fitnet ANN model. The MSE, R and R^2^ of the training, validation, and testing datasets are presented in (Table 3). However, the overall R and R^2^ value was utilized in selecting the best predictive model for each of the response variable. Besides, scatter plots with coefficient of correlation are presented in (Fig. 3).

**Table 3:** MSE, R and R^2^ Values obtained from Model Training, Validation and Testing.

	1ST RUN			2ND RUN			3RD RUN			4TH RUN			5TH RUN		
	MSE	R	R^2^	MSE	R	R^2^	MSE	R	R^2^	MSE	R	R^2^	MSE	R	R^2^
Bulk Density (70 15 15 10 neurons)															
Training	8.77E−06	0.99	0.981	9.87E−06	0.989	0.98	5.58E−06	0.991	0.983	5.36E−06	0.993	0.987	2.35E−05	0.978	0.957
Validation	1.29E−05	0.99	0.98	1.06E−05	0.981	0.962	5.32E−05	0.975	0.951	6.76E−05	0.951	0.904	1.49E−05	0.988	0.978
Testing	3.66E−05	0.956	0.915	2.38E−05	0.977	0.956	3.87E−05	0.965	0.932	4.93E−05	0.819	0.67	5.08E−05	0.97	0.942
Overall		0.985	0.971		0.987	0.974		0.981	0.963		0.962	0.926		0.978	0.956
WABS (70 15 15 10 neurons)															
Training	0.418	0.839	0.705	1.67	0.993	0.987	1.64	0.994	0.988	2.53	0.99	0.98	2.34	0.991	0.983
Validation	0.01	0.905	0.82	9.79	0.979	0.958	6.37	0.971	0.944	6.55	0.981	0.963	3.75	0.988	0.976
Testing	0.025	0.721	0.52	3.24	0.992	0.985	3.91	0.99	0.981	7.43	0.981	0.962	2.67	0.994	0.988
Overall		0.752	0.565		0.989	0.978		0.99	0.981		0.986	0.973		0.999	0.998
Pectin Solubility (70 15 15 5 neurons)															
Training	0.011	0.901	0.813	0.865	0.937	0.879	0.619	0.962	0.926	0.018	0.872	0.761	0.845	0.944	0.892
Validation	9.94	0.999	0.999	0.142	0.991	0.982	0.027	0.932	0.868	0.538	0.875	0.766	0.811	0.959	0.92
Testing	0.013	0.919	0.845	0.019	0.819	0.672	0.67	0.679	0.462	0.019	0.981	0.964	0.011	0.888	0.79
Overall		0.931	0.867		0.934	0.873		0.933	0.869		0.901	0.812		0.936	0.877
Pectin Solubility (70 15 15 8 neurons)															
Training	0.013	0.903	0.816	0.011	0.91	0.828	0.825	0.935	0.874	0.954	0.927	0.86	0.011	0.93	0.865
Validation	0.74	0.956	0.914	0.141	0.998	0.997	0.01	0.966	0.933	0.013	0.947	0.896	0.017	0.91	0.829
Testing	0.021	0.869	0.756	0.483	0.733	0.538	0.997	0.938	0.879	0.292	0.978	0.956	0.532	0.957	0.917
Overall		0.903	0.817		0.936	0.877		0.937	0.879		0.935	0.875		0.918	0.844
Pectin Solubility (70 15 15 10 neurons)															
Training	0.621	0.954	0.911	0.015	0.884	0.781	0.517	0.954	0.911	0.01	0.922	0.851	0.479	0.964	0.929
Validation	0.02	0.844	0.712	0.012	0.916	0.84	0.017	0.92	0.847	0.101	0.957	0.916	0.024	0.893	0.797
Testing	0.021	0.909	0.826	0.032	0.853	0.727	0.017	0.789	0.623	0.01	0.96	0.923	0.01	0.831	0.69
Overall		0.926	0.858		0.873	0.763		0.936	0.877		0.936	0.877		0.938	0.879

**Figure 4:**
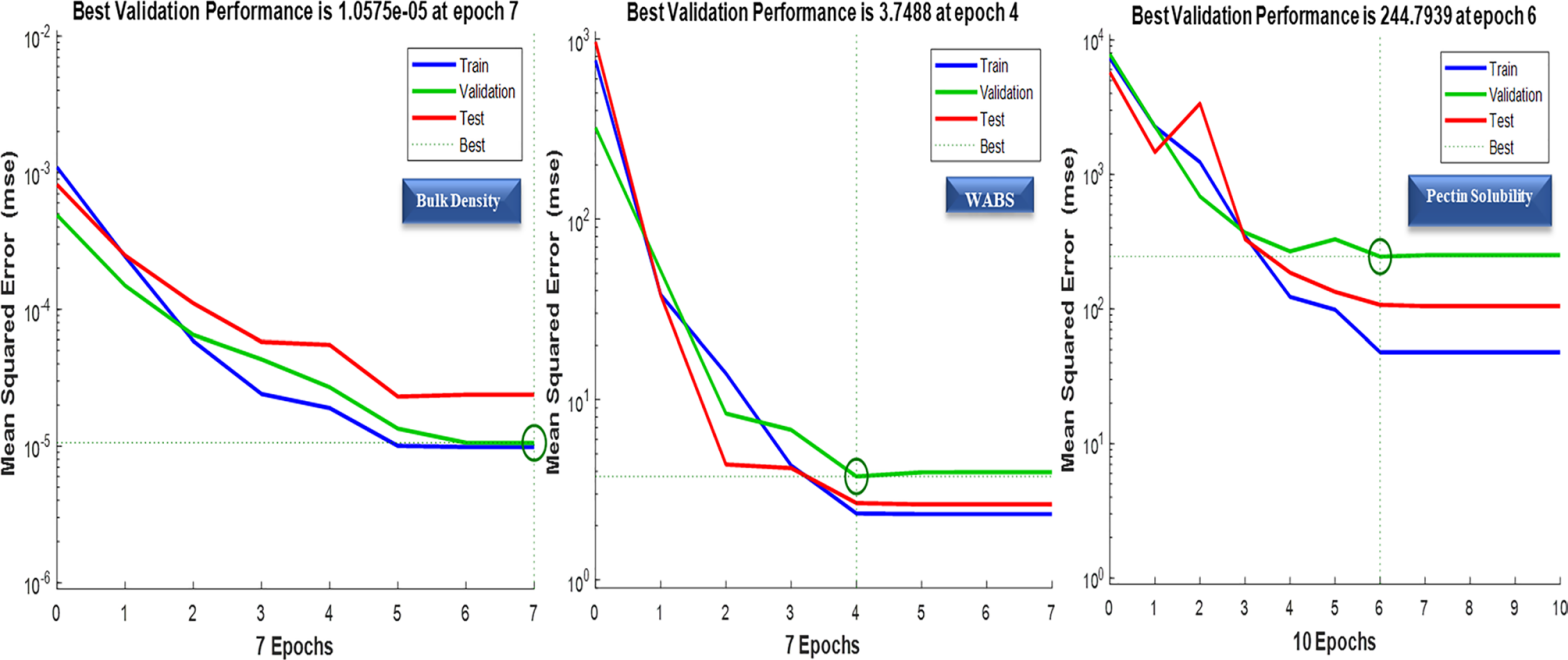
Validation means square error graphs of the chosen models.

**Figure 5:**
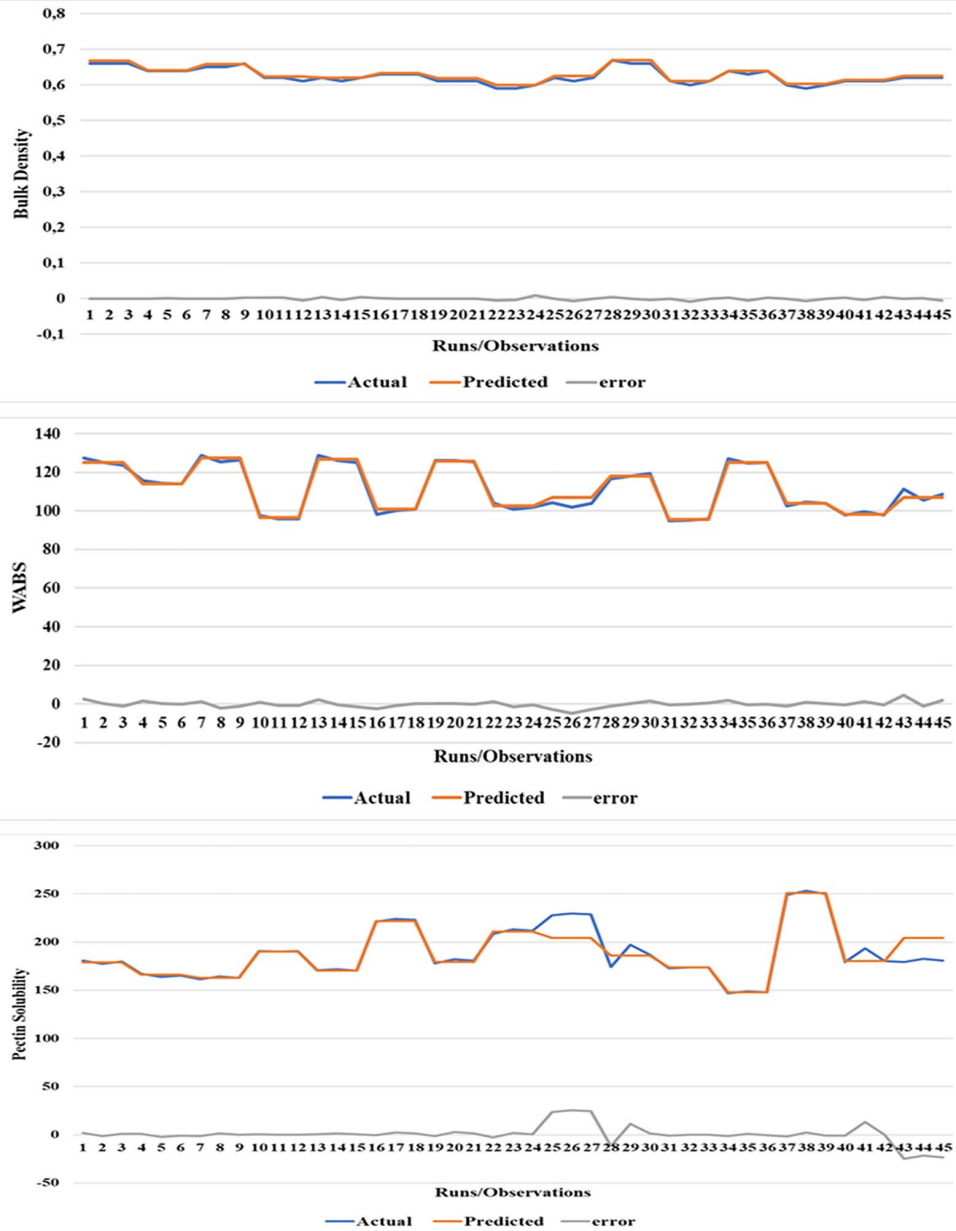
Predicted, actual response, and error for the cooking characteristics studied.

The plot of the best validation performance, together with the actual, predicted response and error (difference between the predicted and actual response) plot are presented in Figs. 4 and 5 respectively. From the evaluation result, for the bulk density obtained after training, validating, and testing the model using 10 neurons, the overall R and R^2^ values are greater than 0.9, implying that ANN was efficient in developing a predictive model for the bulk density of cowpea prepared using infrared heating.

Specifically, the highest overall R and R^2^ values are 0.987 and 0.974 respectively with a validation MSE of 1.06E-05. This was compared with the R^2^ obtained using linear, 2FI and quadratic model (Table 4). Comparatively, the R^2^ of the predictive model generated using ANN was approximately higher by 21%, 13%, and 3% for linear, 2FI and quadratic model respectively.

**Table 4:** R and R^2^ values of Conventional and ANN models in the present study.

Response	Bulk density	WABS	Pectin solubility
Variables	M,T,t	M,T,t	M,T,t
Predictive Models	Present Work	Present Work	Present Work
Linear[R^2^]	0.7700	0.7143	0.3140
2FI[R^2^]	0.8511	0.7653	0.4034
Quadratic[R^2^]	0.9477	0.8864	0.4904
ANN [R]	0.9874	0.9991	0.9380
ANN[R^2^]	0.9750	0.9982	0.8777

M: Moisture, T: Temperature, t: time.

For WABS, the overall R and R^2^ ranged between 0.5 and 0.9991. After repeated training, validation and testing (10 neuron), a significantly high R and R^2^ values were eventually obtained at the 5^th^ iteration with R and R^2^ values of 0.999 and 0.998 respectively with a validation MSE of 3.75 Similar to the bulk density, the R^2^ value was compared with that obtained using linear, 2FI and quadratic model (Table 4). Comparatively, the R^2^ of the predictive model generated using ANN was approximately higher by 28%, 23%, and 11% for linear, 2FI and quadratic model respectively. Studies reported that ANN is an accurate and model with satisfactory prediction. The training, testing and validation results model prediction obtained at about 6–15 neurons, regression of coefficient and lower error in prediction of about 0.9 and 0.02 respectively indicating better ANN prediction of the hydration behavior of green chickpea and soybean seeds at varying soaking temperature and time^24,25^.

For Pectin solubility, the coefficient of regression for the linear, 2FI and quadratic model for pectin solubility are very low, ranging from 0.30 to 0.49, however, using ANN, a high R and R^2^ was obtained after training, validating, and testing the model using three different neurons (5,8 and 10 neurons). The three different neurons employed for pectin solubility were utilized to obtain a significant overall model performance indicator (R and R^2^). The best overall R and R^2^ values were obtained using 10 neurons and at the 5th run, with values of 0.938 and 0.88 respectively, and validation MSE of 245. Comparatively the R^2^ value of the model predicted using ANN was approximately higher by 64%, 54%, and 44% for linear, 2FI and quadratic model respectively. The predictive model developed using ANN are presented in equation. Although there is no report on using ANN for predicting pectin solubility of processed legumes making this finding the first report, however a study using hybrid ANN, RSM and genetic algorithm reported R^2^ value of about 0.94 for predicting percentage protein retention of soybeans subjected to optimization of soaking conditions and considered ANN as alternative to the time-consuming soaking process, extensively practiced in industries, in terms of process time economy^25^.

**Figure 6:**
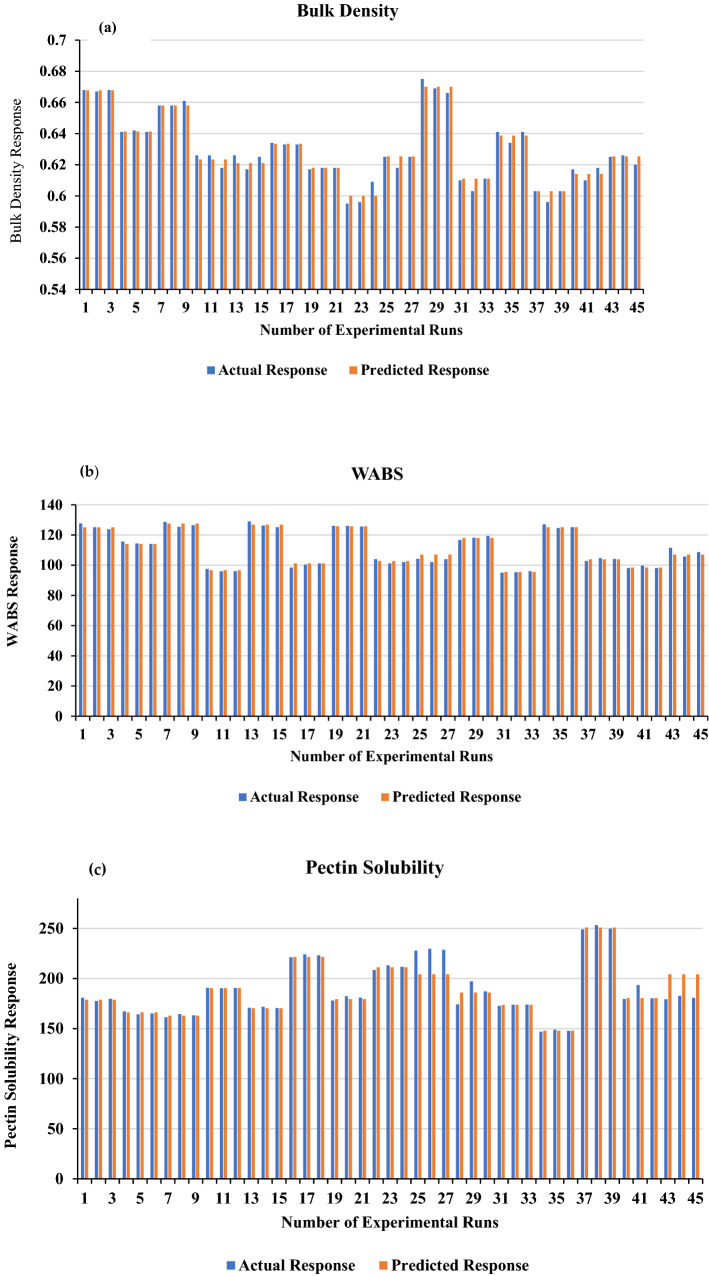
Comparison of actual and predicted response (**a** Bulk density; **b** WABS; **c** Pectin solubility).

The results of comparison between the actual and predicted values as shown in Fig. 6, indicated that the values of the parameters measured in this study (bulk density, WABS and pectin solubility) are closely related. Reportedly, ANN has been utilized in various fields, nonetheless reports on using ANN for predicting the cooking properties of cowpea when moisture content, temperature and time are varied during infrared heating was not found. This study fills that gap by presenting a method of using artificial intelligence technologies (specifically ANN) for predicting the bulk density, WABS and pectin solubility of cowpea precooked via the use of infrared form of heating under varied independent parameters of moisture content, temperature, and time.

1 $${\varvec{y}}_{1} = {\varvec{netX}}$$

2 $${\varvec{y}}_{2} = {\varvec{netX}}$$

3 $${\varvec{y}}_{3} = {\varvec{netX}}$$

4 $$\left[ {\varvec{X}} \right] = \left[ {{\varvec{X}}_{{\varvec{M}}} \user2{ X}_{{\varvec{T}}} \user2{ X}_{{{\varvec{time}}}} \user2{ }} \right]$$

${\text{X comprises of the three dependent variables of moisture}},{\text{ temperature}},{\text{ and time}}$, while ${\varvec{y}}_{1} = \user2{Bulk density }$, ${\varvec{y}}_{2} = \user2{WABS }$, ${\varvec{y}}_{2} = \user2{Pectin solubility }$. net stands for the neural network model developed using ANN after training, validating, and testing raw dataset obtained from the experiment carried out in this study.

## Conclusion

Industry 4.0 epitomizes the fourth wave of industrial revolution and aims at applying computerized, artificial intelligent, and data-driven technologies to research and industrial operations in order to optimize productivity and efficiency. One of these technologies is artificial neural network that works on the principle of self-learning to develop accurate predictive models that can describe and predict the relationship and behavior of a system. For the first time, artificial neural network was utilized in this work to develop a model that predicts the behavior (bulk density, water absorption capacity and pectin solubility) of cowpea prepared using infrared heating). Unlike the traditional linear, 2FI and quadratic model, artificial neural network (ANN proved more accurate and developed a better predictive model in the model prediction with a highly significant model performance (R value of 0.9874, 0.9991 and 0.9380 for bulk density, water absorption capacity and pectin solubility) of infrared-cooked cowpea. The predictive model generated can predict similar response variables subjected to similar or almost-close process parameters (moisture content, temperature, and time) and paves the path for the optimization of the characteristics of infrared-cooked cowpea using artificial intelligence technologies.

## Data availability

The datasets generated during and/or analyzed during the current study is available from the corresponding author on reasonable request.
